# Dr. Piers James Hatton

**Published:** 1911-09-16

**Authors:** 


THE death is announced as having taken place at Carlsbad of Dr. Piers James Hatton, in his forty-seventh year. Dr. Hatton had retired from general practice, and spent much of his time in travelling. He graduated at the University of Edinburgh in 1891 as M.B., C.M., but he also obtained the diplomas of the Royal Colleges in Edinburgh and the Glasgow diploma. He was also a student at the Queen's College, Birmingham.

